# Genome and Transcriptome Analysis of the Fungal Pathogen *Fusarium oxysporum* f. sp. *cubense* Causing Banana Vascular Wilt Disease

**DOI:** 10.1371/journal.pone.0095543

**Published:** 2014-04-17

**Authors:** Lijia Guo, Lijuan Han, Laying Yang, Huicai Zeng, Dingding Fan, Yabin Zhu, Yue Feng, Guofen Wang, Chunfang Peng, Xuanting Jiang, Dajie Zhou, Peixiang Ni, Changcong Liang, Lei Liu, Jun Wang, Chao Mao, Xiaodong Fang, Ming Peng, Junsheng Huang

**Affiliations:** 1 Key Laboratory of Monitoring and Control of Tropical Agricultural and Forest Invasive Alien Pests, Ministry of Agriculture, Environment and Plant Protection Institute, Chinese Academy of Tropical Agricultural Sciences, Haikou, China; 2 Institute of Tropical Bioscience and Biotechnology, Chinese Academy of Tropical Agricultural Sciences, Haikou, China; 3 BGI-Shenzhen, Shenzhen, China; University of Wisconsin - Madison, United States of America

## Abstract

**Background:**

The asexual fungus *Fusarium oxysporum* f. sp. *cubense* (Foc) causing vascular wilt disease is one of the most devastating pathogens of banana (*Musa* spp.). To understand the molecular underpinning of pathogenicity in Foc, the genomes and transcriptomes of two Foc isolates were sequenced.

**Methodology/Principal Findings:**

Genome analysis revealed that the genome structures of race 1 and race 4 isolates were highly syntenic with those of *F. oxysporum* f. sp. *lycopersici* strain Fol4287. A large number of putative virulence associated genes were identified in both Foc genomes, including genes putatively involved in root attachment, cell degradation, detoxification of toxin, transport, secondary metabolites biosynthesis and signal transductions. Importantly, relative to the Foc race 1 isolate (Foc1), the Foc race 4 isolate (Foc4) has evolved with some expanded gene families of transporters and transcription factors for transport of toxins and nutrients that may facilitate its ability to adapt to host environments and contribute to pathogenicity to banana. Transcriptome analysis disclosed a significant difference in transcriptional responses between Foc1 and Foc4 at 48 h post inoculation to the banana ‘Brazil’ in comparison with the vegetative growth stage. Of particular note, more virulence-associated genes were up regulated in Foc4 than in Foc1. Several signaling pathways like the mitogen-activated protein kinase Fmk1 mediated invasion growth pathway, the FGA1-mediated G protein signaling pathway and a pathogenicity associated two-component system were activated in Foc4 rather than in Foc1. Together, these differences in gene content and transcription response between Foc1 and Foc4 might account for variation in their virulence during infection of the banana variety ‘Brazil’.

**Conclusions/Significance:**

Foc genome sequences will facilitate us to identify pathogenicity mechanism involved in the banana vascular wilt disease development. These will thus advance us develop effective methods for managing the banana vascular wilt disease, including improvement of disease resistance in banana.

## Introduction

The species *Fusarium oxysporum* (Fo) comprises a group of ubiquitous inhabitants of soils and plant pathogens causing vascular wilt and root diseases on a broad range of agricultural and ornamental plants worldwide [1]. The plant-pathogenic Fo can be divided into more than 120 formea speciales (f. sp.) according to the pathogenicity to a set of host plants [2], and some formea speciales of Fo are further divided into several physiological races. *F. oxysporum* f. sp. *cubense* (Foc) is the causal agent of fusarium wilt of banana (*Musa* spp.), which is one of the most important constraints on banana production and cause serious economic losses worldwide. It can be divided into four physiological races, race 1, 2, 3 and 4. Race 1 infects the banana cultivars ‘Gros Michel’ (*Musa* sp. AAA group), ‘Pome’, ‘Silk’ and ‘Pisang Awak’ (*Musa* sp. AAB group) and causing the 20th century epidemic. Race 2 infects the cultivar ‘Bluggoe’ and its closely related cultivars. Race 3 does not infect *Musa* species. By contrary, race 4 has a remarkably broad host range infecting almost all cultivars including ‘Dwarf Cavendish’ (*Musa* sp. AAA group) as well as the hosts of races 1 and race 2.

**Figure 1:**
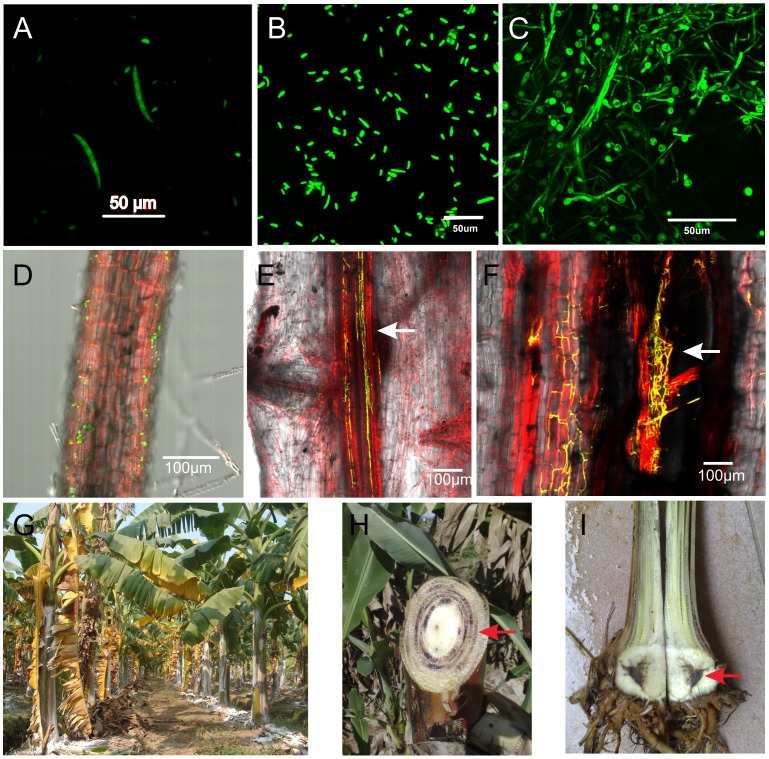
Infection cycle of the banana vascular wilt pathogen *Fusarium oxysporum* f. sp. *cubense* (Foc). (A) macroconidia. (B) microconidia. (C) chlamydospore produced by the GFP-marked Foc isolate. (D) Attachment of Foc hyphae on banana roots. (E) Colonization of Foc hyphae in vascular bundles of banana roots (the arrow indicated). (F) A longitudinal section of banana roots shows fungal hyphae growing in vascular bundles. (G) The diseased banana plants with the dominant symptoms of yellowing leaves. (H–I) the vascular bundles of pseudostem and rhizome from diseased banana turn dark-reddish brown (the arrow indicated).

The asexual fungus Foc produces three types of asexual spores including macroconidia, microconidia and chlamydospore in its life cycle (Figure 1 A–C), enabling it to disperse and survive. It shares a similar infection cycle with *F. oxysporum* f. sp. *lycopercisi* (Fol) causing tomato wilt disease. Firstly, Foc conidia germinate and form fungal hyphae under various nutrients conditions and in the host plant environment. Further, fungal hyphae spread around and colonize at root surface. After that, the fungal hyphae would cross the epidermis (Figure 1 D), and then invade and colonized xylem vessels of root (Figure 1 E & F). After successfully infecting banana roots, the pathogen grows toward the rhizome and pseudo stem, and causes death of tissue or the entire plant. Last, fungal hyphae and spores on the debris of banana plant might fall into soil through rainwater and restart a new infection cycle. Externally, infection and colonization of banana plants by the fungal pathogen always results in wilting and yellowing of the lower part of leaves (Figure 1 G). Internally, discoloration of rhizomes and necrosis of vascular bundles in pseudo stem can be observed in seriously infected banana plants (Figure 1 H–I, arrows indicated).

As a saprophyte, Foc can persist in soil for a long time. Once it recognizes and perceives the cues from host plants, it begins infecting host bananas from roots. Few effective options for managing this ineradicable pathogen, as fungicides are largely ineffective [3]. Therefore, formulating effective control methods for fusarium wilt of bananas is a thing of great urgency, and this largely requires better understanding of the fungal pathogen, especially its genome. Previously, the genomes of the tomato pathogen Fol and the maize pathogen *F. verticillioides* were sequenced and the Fusarium comparative genomics highlights the lineage-specific genomic regions in Fol that are responsible for the polyphyletic origin of host specificity [4]. In the present study, our objectives are: (1) Sequencing and analyzing the genomes of the isolates N2 (race 1, Foc1) and B2 (race 4, Foc4) of Foc. (2) Exploring the putative virulence associated genes. (3) Analyzing the transcriptomes of Foc1 and Foc4 at both vegetative growth stage and 48 h post inoculation to the Cavendish banana ‘Brazil’. All of these will facilitate identification of the pathogenicity mechanisms involved in vascular wilt development, and molecular mechanism underlying the difference in virulence between Foc1 and Foc4.

## Results and Discussion

### Genome Sequencing and General Features

The isolates N2 (race 1, Foc1) and B2 (race 4, Foc4) of Foc were each sequenced to at least 106×coverage (Table1 and Table S1 in File S1). The genome of Foc1 was assembled into 461 scaffolds (>2 kb; N50, 653.1 kb) containing 2,977 contigs with a total size of 47.84 Mb. The genome of the isolate B2 was assembled into 164 scaffolds (>2 kb; N50, 1.9 Mb) containing 4,109 contigs with a total size of 53.12 Mb (Table 1 & Table S2 in File S1). The assembly sizes of both Foc isolates resemble that of Foc tropical race 4 strain II5 (46.55 Mb), which was released by Broad institute (http://www.broadinstitute.org/annotation/genome/fusarium_group/). Foc1 and Foc4 were predicted to have 17,462 and 18,065 coding genes, respectively. The coding capacities are thus similar to those of other ascomycetes such as Foc strain II5 (16,634) and Fol strain 4287 (20,925) [4].

**Table 1:** Features of the *F. oxysporum* f. sp *cubense* race 1 and race 4 genomes.

Features	Foc1	Foc4
Genome size (bp)	47,838,384	53,119,146
Coverage (fold)	106×	132×
G+C content (%)	47.98	48.05
Protein coding genes	17,462	18,065
*RPKM>1 (genes)	11,484	12,763
*RPKM>5 (genes)	8,467	9,971
Coding region (bp)	25,643,169	28,419,821
Percent coding (%)	53.6	53.5
Exon number	49,212	50,991
Exon length (bp)	22,414,389	23,357,730
Mean exon length (bp)	455.46	458.08
Exon number/gene	2.82	2.82
InterPro signature	11,205	11,591
GO assignment	8,734	9,016
KEGG alignment	3,614	3,743
Swissport alignment	9,777	10,153
Trembl alignment	15,607	16,170
Total annotations	15,692	16,288

*RPKM: Reads Per Kb per Million reads, which is used to estimate expression levels of genes.

The 5 Mb difference in genome size between Foc1 and Foc4 is probably due to the fact that we constructed more libraries with large inserts and got more mate pair information for Foc4, which benefited to connect contigs into scaffolds for Foc4 but meanwhile introduced more gaps (Table S1 in File S1). To check the integrity of the assembly, sequenced reads were aligned to the corresponding assemblies, about 98.05% of Foc1 reads from libraries with small size inserts can be aligned to the Foc1 assemblies, and 97.96% of Foc4 reads can be aligned to the Foc4 assemblies. High map ratio indicates that the assemblies represent most of the genomes.

In both Foc1 and Foc4 genomes, coding regions account for ∼53.50% of the genome with 2.82 exons per gene; the average exon length is 480 bp. Moreover, a bidirectional best hits (BBH) analysis revealed that Foc1 and Foc4 genomes shared 15,140 orthologs with average 96.7% of amino acid identity. 15,692 (or 90.0%) coding genes in Foc1 and 16,288 (or 90.2%) coding genes in Foc4 were functionally annotated after alignment of these sequences to the known databases including Gene Ontology (GO), Kyoto Encyclopedia of Genes and Genomes (KEGG) [5], SwissProt and TrEMBL [6] (Table 1). Interestingly, of 15,140 orthologs shared by Foc1 and Foc4, 129 showed poor amino acid identity (<50%) (Table S3). Only one third of these orthologs (37 genes in Foc1 and 41 in Foc4) could be categorized into some functional groups based on GO terms, and no more than one half of these genes have annotations in KEGG and Interpro databases, indicting these genes might be under rapid evolution and thus displayed high variations.

### Differences in Genome Structure between Foc and Fol

**Figure 2:**
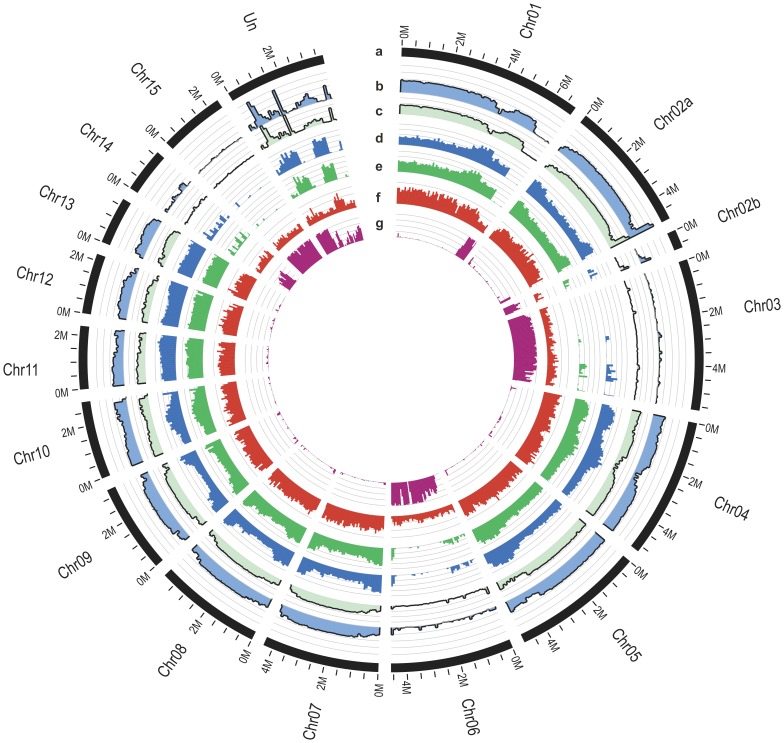
Comparison of genome structures of the *F. oxysporum* f. sp. *cubense* race 1 (Foc1) and race 4 (Foc4) with that of *F. oxysporum* f. sp. *lycopersici* (Fol). The size of window is 100(a) *F. oxysporum* f. sp. *lycopersici* (*Fol*) chromosomes. (b) The average depth of Foc1 reads mapped on Fol chromosomes. (c) The average depth of Foc4 reads mapped on Fol chromosomes. (d) The SNP and InDel number of Foc1. (e) The SNP and InDel number of Foc4. (f) Gene density of Fol. (g) Repetitive sequence density of Fol.

The comparative analysis of Fusarium genomes conducted by Ma L *et al*. revealed that the Fol possesses lineage-specific (LS) genomic regions including four entire chromosomes (Chr03, Chr06, Chr14 and Chr15), which are rich in transposons and genes related to pathogenicity [4]. To confirm presence of the LS genomic regions in Foc, all sequence reads of Foc1 and Foc4 were aligned against the Fol genome using BWA with parameters including sequence similarity, pair-end relationships and sequence quality. We found that the assemblies of Foc1 and Foc4 can be mapped to the most chromosomes of Fol, except four chromosomes (Chr03, Chr06, Chr14 and Chr15, Figure 2). Therefore, similar to the genomes of *F. graminearum* and *F. verticillioides* [4], both Foc genomes contain no the lineage-specific (LS) genomic regions that unique to Fol. This supports that the four chromosomes in Fol are the lineage-specific regions.

### Gene Families and Phylogenetic Relationship of Some Sequenced Fusaria

**Figure 3:**
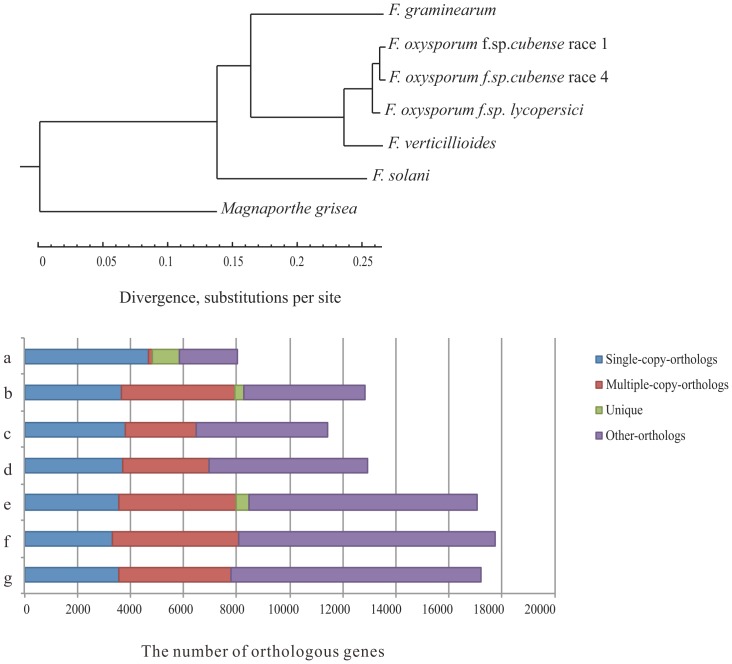
The orthologs and phylogenomic relationships of both *F. oxysporum* f. sp. c*ubense* isolates and other sequenced fungi. (**A**) The phylogenetic tree of the seven sequenced fungi. (**B**) Statistics of orthologs in the seven sequenced fungi including *Fusarium graminearum* (a), *F. oxysporum* f. sp *cubense* race 1 (b), *F. oxysporum* f. sp *cubense* race 4 (c); *F. oxysporum* f. sp *lycopersici* (d), *F. verticillioides* (e), *F. solani* (f), *Magnaporthe grisea* (g).

17,196 (98.5%) of Foc1 genes can be assigned into 12,366 gene families at least with one other species, while 17,758 (98.3%) of Foc4 genes can be assigned into 12,365 gene families. 2,997 single-copy gene orthologous groups were obtained, and sequences of single-copy genes from each species were concatenated into a super gene to infer the phylogeny tree. As shown in Figure 3, the tree indicates a close relationship between two sequenced Foc isolates and Fol, suggesting that they might descent from a common ancestor.

### Virulence Associated Genes

To find potential virulence associated genes, a whole genome BLAST analysis was conducted against the pathogen-host interaction (PHI) gene database (http://www.phi-base.org/), a collection of genes proven to affect the outcome of pathogen-host interactions from fungi, oomycetes and bacteria [7]. After removing the genes that are not related to pathogenicity, we identified 347 and 348 putative virulence associated genes (VAGs) in Foc1 and Foc4, respectively. The number of VAGs in Foc1 and Foc4 is comparable to that in the Foc race 4 strain II5 (357) and other sequenced fungi (Table S4 & Table S5 in File S1). The search of the PHI database yielded 16 already characterized VAGs from Fo, including *ARG1* [8], *CHS2* [9], *CHSV* [10], *CHS7* [9], *FMK1* [11], *FGA1* [12], *FGA2* [13], *FGB1* [14], *FOW1* [15], *FOW2* [16], *foSNF1* [17], *FRP1* [18], *GAS1* [19], *PacC* [20] and *SIX1* [21], indicating conservation in VAGs and pathogenesis among the different formae speciales of Fo. A number of Foc orthologs were found in the cereal pathogenic fungi *F. graminearum* (13 genes), *Magnaporthe oryzae* (88) and *Ustilago maydis* (31). Also, 23 Foc genes are similar to the characterized VAGs of the necrotrophic fungus *Botrytis cinerea*. Interestingly, numerous Foc orthologs were found in animal pathogenic fungi such as *Cryptococcus neoformans* (57) and *Candida albicans* (99), which could be explained by the fact that Foc lacks infection structures (i.e. appressoria) during host penetration, which is similar to both the animal pathogens.

### Gene Involved in Adhesion to Host

The ability of fungi to adhere to target tissue could attribute to pathogenicity. Deletion of the adhesin gene WI-1 (related BAD1, PHI: 135) from *Blastomyces dermatitidis* resulted in impairment in binding and entry of yeasts into macrophages, loss of adherence to lung tissue and virulence in mice [22]. Similar functions were found in SOWgp (PHI: 272) from *Coccidioides immitis* [23]. Deletion of the MAP Kinase gene *fmk1* result in impaired root attachment in Fo [11]. Prados-Rosales R *et al.* identified a fraction of proteins involved in attachment of roots from cell wall proteome of Fol, but these proteins lacked functional information [24]. Here, we identified 5 and 6 adhesin genes in Foc1 and Foc4 genomes, respectively,one of which is the counterpart of SOWgp. While, the II5 strain genome of Foc race 4 contains the similar sequences of both WI-1 and SOWgp. Notably, almost all putative adhesion genes of Foc (5 in Foc1 and Foc4) had mRNA transcripts at both vegetative growth stage and 48 h post inoculation to the banana ‘Brazil’. Moreover, 3 adhesin genes were markedly (above 3-fold) induced in Foc4 banana, but only one was up regulated in Foc1 at 48 h post inoculation to the banana ‘Brazil’ in comparison with the vegetative growth. These suggest that adhesin genes might play roles during the fungal pathogen was exposed to the banana ‘Brazil’.

### Secreted Proteins

Both Foc isolates encode ∼1300 of putative secreted proteins (SPs, 1298 for Foc1 and 1342 for Foc4), accounting for appropriately 7.4% of all predicted proteins. The numbers are comparable to that for *F. fujikuroi* (1,336), but fewer than that reported for *F. graminearum* (1,442) [25] and Fol (1,541) [4]. A set of secreted carbonhydrate-active enzymes (CAZys) encoded by both Foc isolates are markedly similar to some characterized virulence factors such as bcpg1 [26], CPPG2 [27], BcPG2 [28] and PELD [29] of pectin lyase (PL) family, xylanase (XYN11A [30]), and beta-1,6-glucanase (VfGlu1 [31]) of glycoside hydrolase family in fungal pathogens (Table S4 in File S1). Intriguingly, Foc4 has more secreted cutinases (11, carbohydrate esterase (CE) family) in comparison with Foc1 (9), three of which show significantly similar to the extracellular cutinase PBC1 [32] from *Pyrenopeziza brassicae*, CUT2 from *M. oryzae* and CutA from *F. solani* f. sp. *pisi,* which are required for the pathogenicity or full virulence on host plants [33]–[36]. Likewise, Foc1 encodes 16 secreted lipases (CE family) versus 18 in Foc4. Of these lipases, one is significantly similar to the lipase FGL1 from *F. graminearum* that is a pathogenicity factor during infection of cereals [37]. These imply that both Foc1 and Foc4 possess a variety of arsenals of secreted CAZys that can be adopted during infection of banana.

Aside from these secreted CAZys, three putative SPs encoded by Foc are significantly homologous to those effectors from the oomycetes *Phytophthora sojae* and *P. infestans* such as INF2A, INF2B [38] GIP1, GIP2 [39], and PosjNIPw [40] (Table S4 in File S1), which are able to elicit hypersensitive response or induce necrosis in host plants, suggesting that these SPs could be involved in Fo-banana interaction. Similarly, some gene products (Table S4 in File S1) are orthologous to the characterized SPs such as PEP1, PEP2, and PEP5 from *Nectria haematococca* (anamorph: *F. solani*) [40], [41], MSP1 [42] and AVR-Pita [43] from *M. oryzae* that contribute to the fungal pathogenicity to pea or rice, indicating that these SPs encoded by Foc may be related to the virulence to banana.

In airborne fungal pathogens, a number of secreted hydrophobins have pleiotropic functions including attachment of spores to hydrophobic surfaces [44], involvement in surface interactions during infection-related development [45], and preventing immune recognition [46]. Although none of hydrophobins in Fo have been characterized, we identified several class II hydrophobins in Foc (3 for Foc1 and 4 for Foc4), one of which is evolutionary related to the hydrophobin MHP1 (PHI: 458) that is essential for fungal development and plant infection by *M. grisea* [47]. Interestingly, relative to that at vegetative growth stage, this hydrophobin gene was dramatically induced in Foc4 at 48 h post inoculation to ‘Brazil’ banana. Conversely, it was suppressed in Foc1, indicating it may be specifically involved surface interactions between Foc4 and banana.

Recent researches on Fol, the causal agent of Fusarium wilt of tomato, have elucidated the roles of some SPs in pathogenicity in the Fol-tomato pathosystem [21], [48]–[53]. The SIX (secreted in xylem) proteins SIX1 (Avr3), SIX3 (Avr2) and SIX4 (Avr1) function as either Avr protein (effector) involved in the incompatible interaction or virulence factors implicated in the compatible interactions between tomato and Fol [21], [48]–[54]. We searched across the Foc1 and Foc4 assemblies to identify the orthologs of these SIX-coding genes (namely *SIX1*–*SIX8*). Our analysis revealed that three orthologs of *SIX1* interspersed in Foc4 genome (namely *Six1a–Six1c*, Table 2), while only one copy of *SIX1* existed in Foc1. Besides, Foc4 has one copy of *SIX2*, *SIX6* and *SIX8*, whereas Foc1 merely has one copy of *SIX6*. This differentiates from the previous study on *SIX* genes using hybridization analysis and PCR [49], which reported one copy of *SIX1*, *SIX7* and *SIX8* in another race 4 isolate of Foc. Since we have manually checked the sequencing depth and synteny relationship nearby these regions, we excluded the possibility of assembly errors and assumed that the difference is probably due to strain variations and some other unknown reasons. Further, we used the same pipeline and parameters to search against the strain II5 genome of the Foc race 4, which also demonstrated the similar results with 3 copies of *SIX1*, one copy of *SIX2* and *SIX6* but without *SIX8* (data not shown) in the strain II5, indicating that three copies *Six1* genes may be conserved in different isolates of Foc and could be involved in pathogenicity against banana.

**Table 2:** The orthologs of *SIX*-genes in Foc1 and Foc4.

SIX genes	Foc1 gene ID	RPKM_0h	RPKM_48h	Foc4 gene ID	RPKM_0h	RPKM_48h
*SIX1a*	Foc1g01632	5.75	39.16	Foc4g00240	6.27	12.77
*SIX1b*	NA	–	–	Foc4g00324	0.49	0
*SIX1c*	NA	–	–	Foc4g00575	50.71	113.08
*SIX2*	NA	–	–	Foc4g07631	NA	NA
*SIX6*	Foc1g00211	14.32	13.31	Foc4g00351	243.71	192.59
*SIX8*	NA	–	–	Foc4g00520	0.16	0.63

Expression of three copies of *SIX1* from Foc4 and one copy from Foc1 was detectable at both the vegetative growth stage and 48 h post inoculation to banana (Table 2), implying they functioned at both stages, and additional copies may contribute to higher pathogenicity of Foc4 to banana. Interestingly, expression of *Six1* gene by Foc1 and Foc4 was induced at pre-infection stage relative to that at vegetative growth stage. This consists with the result that *SIX1* was induced immediately upon penetration of the root cortex in tomato, and induction required living plant cells [52]. Additionally, Foc4 contains *SIX2* and *SIX8* genes that are absent in Foc1, we thus further assume that *SIX2* and *SIX8* genes may have roles in infection of Cavendish banana ‘Brazil’ and contribute to the broader host range of Foc4. Further assays including gene deletion and complementary may better demonstrate the functions of these genes in Foc4.

### Genes Putatively Involved in Detoxification and Transportation

Plants produce various secondary metabolites, many of which have antifungal activity, such as saponin, flavone and cyclohexenone [55], and these antifungal molecules may provide a preformed chemical barrier against phytopathogenic fungi. Correspondingly, fungi have evolved a diversity of enzymes to detoxify toxins. We revealed some sequences that resemble the PHI sequences in both Foc genomes such as GzmetE (PHI: 355) from *F. graminearum*, tomatinase (tom1, PHI: 191) from the tomato pathogen Fol as well as the kievitone hydratase ‘khs’ and flavin-containing mono-oxygenase MAK1 (PHI: 112) from *F. solani*. Both GzmetE and tomatinase were characterized to participate in detoxification of saponin [56], [57]. Also, both khs and MAK1 are capable to catalyze conversion of the antimicrobial phytoalexins ‘kievitone’ and ‘maackiain’ to less toxic metabolites [58], [59], respectively. These imply that Foc might produce a variety of enzymes that participate in the detoxification of antifungal molecules that preformed in host banana during its colonization.

Cytochrome P450s (CYP) play essential roles in the fungal biosynthesis of secondary metabolites and detoxification of toxic compounds [60]–[64]. Both Foc genomes encode a great number of putative CYP genes (173 CYPs in Foc1 and 176 in Foc4, Table 3), some of which are similar to the characterized fungal CYP sequences including the monooxygenase gene *BcBOT1* (PHI: 438) and the demethylases genes *PDAT9* and *PDA6-1*, the products of which are either involved in phytotoxin biosynthesis in *Botrytis cinerea* (BcBOT1) [65], or participate in detoxify the phytoalexin pisatin from garden pea (PDAT9 and PDA6-1) [63], [66]. Additionally, the orthologs of the sterol 14alpha-demethylase enzyme genes *MoCYP51*A and *MoCYP51B* that are essential for virulence to rice in *M. oryzae* [67], were also discovered in both Foc genomes.

**Table 3:** Protein classification of Foc1 and Foc4.

Protein classification	Foc1	Foc4
Carbonhydrate-active enzymes	514	515
Cytochrome P450	173	176
G protein	5	5
G protein coupled receptor	19	17
Histidine kinase	20	20
Protein kinase	94	103
Peroxidases	25	28
Pth11 like GPCR	94	92
Secondary metabolite backbone genes	32	34
Transcription factor	729	793
Transporter	1001	1040

Besides the detoxification enzymes, some peroxidases were putatively implicated in detoxification of host defense metabolites. Foc1 encodes 25 peroxidases versus 28 for Foc4 (Table 3). 3 proteins encoded by Foc are highly similar to the catalase-peroxidases VlcpeA [68] from *Verticillium longisporum* and CPXB [69] from *M. oryzae* that have roles in defense against hydrogen peroxide (H_2_O_2_) generated by the host plant during the fungal infection. Additionally, we also found the Foc homologs of the glutathione peroxidase HYR1 [70] that has similar functions in *M. oryzae*.

Different transporters are essential for import of the nutrients and export of secondary metabolites and other toxic compounds. Both Foc genomes encode a large number of transporters (1001 in Foc1 and 1040 in Foc4,Table 3). The major facilitator superfamily (MSF) and the amino acid-polyamine-organocation (APC) family were preferentially expanded in Foc4 (405 MSF, 100 APC) relative to Foc1 (379 MSF, 91 APC). MSF proteins were usually involved in the transport of a wide range of substrates [71]–[73], and APC transporters mediate uptake of amino acids and their derivatives [74]. The enrichment of both the families in Foc4 implies that Foc4 might have a greater ability to access a range of nutrients than do Foc1. Interestingly, 16 of these putative transporters from both Foc1 and Foc4 are similar to the virulence-associated proteins in PHI database (Table S4 in File S1) including 8 in MSF, 5 in ATP-binding cassette (ABC) superfamily and 3 in the p-type ATPase (p-ATPase) superfamily. The transporter CFP that belongs to MSF was characterized to facilitate transport of phytoxins and involved in toxin secretion, and the five members of ABC (ABC1, ABC3, GPABC1, BcatrB and MgAtr4) are required for export of fungitoxic compounds [75]–[80]. These indicate that transporters of MSF, ABC and p-ATPase families might be implicated in export of antifungal compounds and be required for Foc virulence.

### The Gene Clusters Involved in Biosynthesis of Secondary Metabolites

Fungi, especially soil-dwelling filamentous fungi, produce an abundant array of secondary metabolites (SMs) including mycotoxins, antibiotics and pharmaceuticals. This impressive amount of SMs provides protection against various environmental stresses and during antagonistic interactions with other soil inhabitants or a eukaryotic host [81]. Recently, some SMs including beauvericin, gibberellins (GAs) and other SMs were found to play important roles in fungus-host interaction. Deletion of a beauvericin synthetase coding gene- *beas* resulted in marked reduction on production of beauvericin, and therefore the mutant demonstrated an attenuated virulence to tomato [82]. Interestingly, beauvericin and fusaric acid were found to be toxins produced by Foc during invasive growth in banana. Both toxins were detected in the all tissues of banana with fusarium wilt symptoms including pseudostems, fruit and leaves, and the contents in banana roots were well correlated with virulence of the isolates of Foc [83]. GA produced by the rice pathogen *F. fujikuroi* accounts for ‘bakanae’ disease of rice. Wiemann P *et al.* revealed that GA biosynthesis genes are present in some related species, but GA synthesis is limited to *F. fujikuroi.* Also, they found the SM product of the PKS19 cluster that is unique to *F. fujikuroi* plays a special role during rice infection [84].

In the present study, 32 and 34 backbone genes were identified in the assemblies of Foc1 and Foc4, respectively (Table 3 and Table S6 in File S1). 2 backbone genes are unique in Foc1 versus 4 in Foc4. We also identified putative 26 and 30 gene clusters involved in the biosynthesis of secondary metabolites for Foc1 and Foc4, respectively (Table S7 & Table S8 in File S1). Functions of the majority of SMs derived gene clusters are largely unknown, some of the gene clusters were putatively involved in the biosynthesis of secondary metabolites including beauvericin, Fusaric acid, Fusarin C, Fumonisin and Fusarubin (Table S6 in File S1). Moreover, a number of putative SMs backbone genes in Foc are orthologous to the virulence-associated genes that were experimentally proven to be involved in secondary metabolites in other fungi. For instance, Foc1g11839 and Foc4g01275 are homologous to the polyketide synthases ALB1 (PHI: 101) and other four homologs (PHI: 40, 116, 433 & 238, Table S4 in File S1) that was characterized to be involved in regulation of virulence of *Aspergillus fumigatus*, *Cercospora nicotianae*, *Colletotrichum lagenarium* and other fungal pathogens [85]–[87]. Similarly, Foc1g07907 and Foc4g04228 are homologous to the avirulence protein ACE1 (PHI: 325) that is involved in secondary metabolism in *Magnaporthe grisea* [88]. We also found that Foc1g06330 and Foc4g02522 are homologs of the cyclic peptide synthetases HTS1 (PHI: 12) from the maize pathogen fungus *Cochliobolus carbonum* [89] and AMT (PHI: 160 ) from *Alternaria alternata* apple pathotype [90] that is involved in AM-toxin synthesis and pathogenicity. Additionally, Foc1g07090 and Foc4g09766 resemble the nonribosomal peptide synthetase NPS6 (PHI: 416, 1008, 1009), which was characterized to be a conserved virulence determinant of plant pathogenic ascomycetes [91]. Together, the presence of these genes in both Foc genomes implies they might be virulence determinants and play roles in Foc-banana interactions.

### Signal Conduction

In order to establish disease, fungal pathogen needs to respond appropriately to the plant environment. In pre-infection course, perception of the signals from the host plant environment is mediated by the receptor on the surfaces of pathogen cells, though a major of receptors remain unknown [92]. Important progress on G protein mediated signaling revealed that G proteins control fungal growth, development and pathogenicity [93]. A novel class of GPCR typified by PTH11 was found to be required for pathogenicity in the plant pathogenic fungus *M. grisea* [94]. Also, GprD in *Aspergillus fumigatus* was suggested as an essential regulator of colony growth, hyphal morphogenesis, and virulence [95]. In Fo, the roles of GPCRs in G protein signaling pathway were not characterized, but G protein alpha and beta subunit were elucidated to be involved in growth, development and pathogenicity (FGA1 [12], FGA2 [13], FGB1 [14]). We revealed ∼115 genes encoding the putative GPCRs (including Pth11 like) and G proteins in Foc (Table S9 in File S1). Both Foc1 and Foc4 have three genes encoding alpha subunit of G protein, two distinct genes encoding beta and gamma subunits. These indicate conservation in G protein signaling pathway among different ascomycete fungi.

The two-component signaling pathways are involved in environmental stress responses, hyphae development, sensitivity to fungicides and virulence in fungi [96]–[106]. Two conserved components are required in these pathways: a histidine kinase (HK) that autophosphorylates in response to an environmental stimulus, and a response regulator (RR) that transmits the signal, resulting in activation of transcription or a mitogen-activated protein kinase cascade [107]. Both Foc1 and Foc4 have 20 putative HKs, 4 of which resemble the virulence associated proteins in PHI database such as FOS1 (PHI: 253) [108], [109], ssrA (PHI: 553) [110], CaSLN1 (PHI: 140) [111] and BOS1 (PHI: 550) [112] as well as its homologs (Table S4 in File S1). Remarkably, Fhk1, the homolog of the histidine kinase BOS1 in *F. oxysporum* was found to be involved in modulating stress adaptation and virulence [113]. Besides, Foc1 and Foc4 encode 3 response regulators that are similar to SSK1 (PHI: 189) [114], SKN7 (PHI:380) [115] and MoRim15 [116] (Table S4 in File S1). SSK1 and SKN7 were characterized to contribute to osmolarity stress and fungicide action in *C. albicans* and *M. oryzae* [99], [115], [116]. SSk1 and MoRim15 are essential for virulence in *C. albicans* and *M. oryzae* [114], [116], respectively.

Protein kinases are responsible for the phosphorylation of proteins, which thus play pivotal roles in signal transduction in eukaryote cells [117]. Both Foc genomes encode ∼100 protein kinases (94 for Foc1 and 103 for Foc4, Table 3 & Table S10 in File S1), 19 of which have highly similar sequences in PHI database (Table S4 in File S1). This implies that protein kinases and the pathways that they are involved in have crucial roles during infection of banana. Among these 19 protein kinases, Fmk1 is unique protein kinase that has been functionally characterized in Fo [11]. The RNA-seq data revealed the transcript levels of some virulence associated kinase genes (7 out of 25) were significantly increased in Foc4 but were decreased or had no variation in Foc1 during exposed to the ‘Brazil’ banana for 48 h as compared to that at the vegetative growth stage. We thus inferred that the seven protein kinases might participate in infection process and contribute to virulence to the banana ‘Brazil’. Unexpectedly, the expression of *Fmk1* was induced neither in Foc1 nor in Foc4, implying that its induction might be not required for Foc during pre-infection of banana.

Following the signal transduction, different transcription factors (TFs) would be activated to regulate physiological response of cells. Foc1 encodes 729 putative transcription factors compared to 793 for Foc4 (Table 3). The numbers of TF families of homeodomain-like and Zn2Cys6 were significantly higher in Foc4 than in Foc1 (Table S11 in File S1). Sixteen of these putative transcription factors have homologs in PHI database (Table S4 in File S1). For example, four putative Zn(II)2Cys6-type transcription factors are markedly similar to Fow2 (PHI: 734), CLTA1 (PHI: 169), MGG_09263 (PHI: 889) and CTB8 (PHI: 1050) that were experimentally proven to be implicated in pathogenicity [16], [118]–[120]. Also, four putative basic-leucine zipper (bZIP) transcription factors in Foc are homologous to the virulence associated proteins including ZIF1 (PHI: 444) from *F. graminearum* [121], YAP1 (PHI: 853) from *Ustilago maydis* [122], CPCA (PHI: 340) from *Aspergillus fumigatus* [123] and CPTF1 (PHI: 344) from *Claviceps purpurea* [124]. Most importantly, we found that Foatf1 is the homolog of YAP1 in Foc4, which is involved in pathogenesis by regulating the oxidative stress responses of Cavendish banana (*Musa* spp.) [125]. Fost12, the Fo ortholog of the yeast homeodomain transcription factor Ste12p, was also characterized to govern invasion growth and pathogenicity [126]. The other virulence-associated genes including SPT3 (PHI: 273), BWC1 (PHI: 430), BWC2 (PHI: 431), MGG_00692 (PHI: 776), RUM1 (PHI: 187), FKH2 (PHI: 252) and MIG1 (PHI: 1070) have counterparts in Foc, implying that they might participate in regulation of the fungal virulence.

Inspecting the expression profiling of different transcription factor genes, 12 out of 16 virulence-associated genes were significantly induced and none was markedly suppressed in Foc4 at 48 h post inoculation to the banana ‘Brazil’ relative to that at vegetative growth stage. In contrast, only 2 of those were induced, and 6 were apparently suppressed in Foc1 (Table S4 in File S1). These suggest that Foc4 could employ more TFs that are associated with virulence as compared to Foc1 during exposed to the banana ‘Brazil’.

### Comparative Transcriptome Analysis

It is well known that the Cavendish banana is resistant to Foc race 1 but is susceptible to Foc race 4. The mechanism underlying the difference in the pathogenicity to Cavendish banana between Foc race 1 and Foc race 4 is still ambiguous. To identify genes and signaling pathways involved in pathogenesis and explore molecular basis of the difference in virulence between two races, we analyzed the transcriptional responses of Foc1 and Foc4 using RNA-Seq. The data generated from Foc1 and Foc4 collected at vegetative stage was used as the control, and the time-point (48 hours post inoculation) was chosen to focus on the crucial pre-infection processes, including adhesion to roots, recognition of host and production of infectious mycelia.

**Figure 4:**
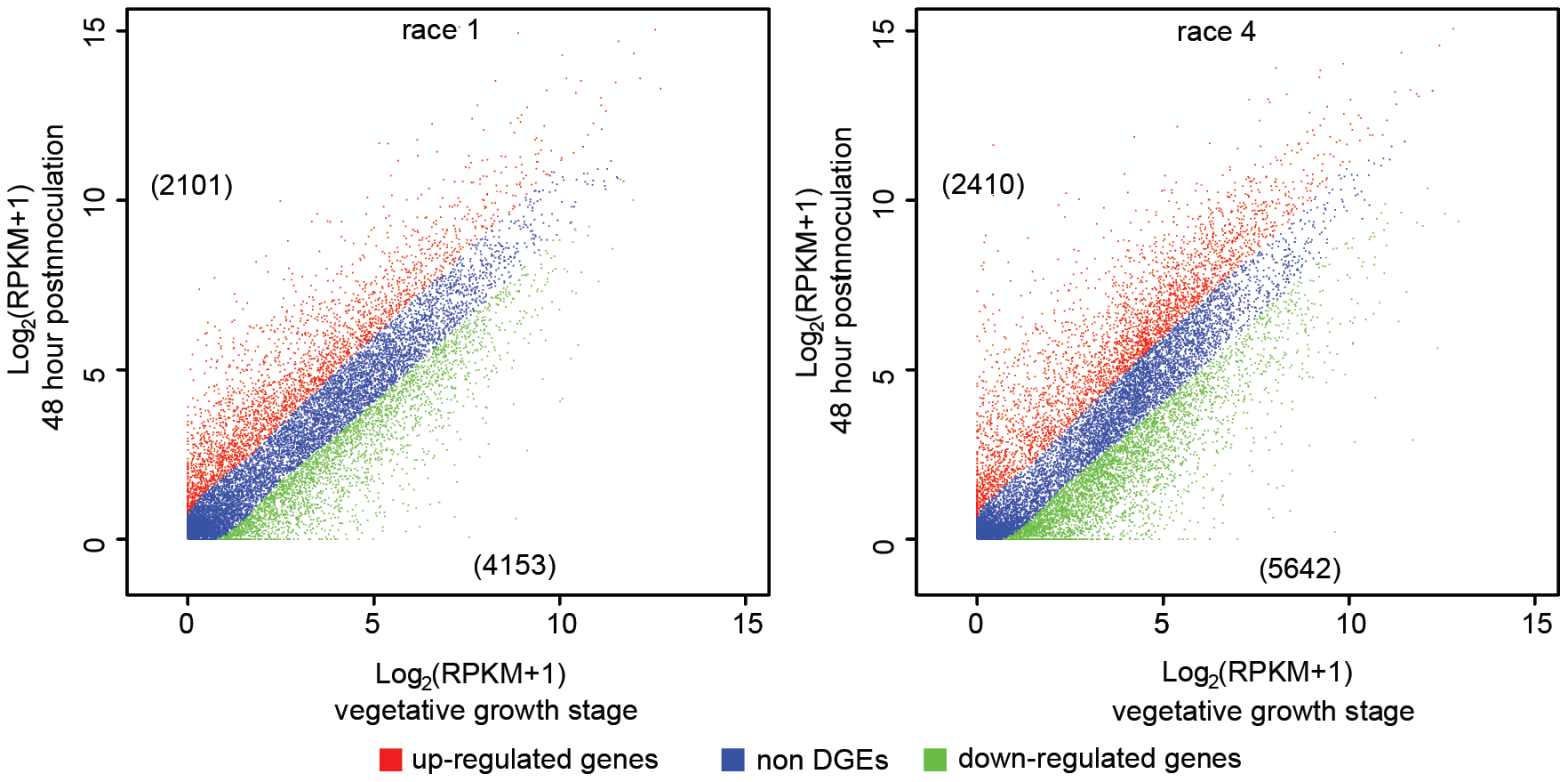
Differential gene expression by *Fusarium oxysporum* f. sp. *cubense* race 1 (Foc1) and race 4 (Foc4) at 48 h post inoculation to the banana ‘Brzail’. Genes differentially expressed by *Fusarium oxysporum* f. sp. *cubense* race 1 (A) and race4 (B) at 48 h post inoculation to the banana ‘Brzail’ (48 h) relative to those at vegetative growth stage (0 h). The figures in parentheses are the number of genes significantly up- or down-regulated by each fungus.

After sequencing, ∼9.2 Gb and 6.4 Gb of sequence data for Foc1 and Foc4 were generated, respectively. 62.5 Mb (∼90.3% of 69.2 Mb) and 62.6 Mb reads (∼91% of 68.6 Mb) can be located on the genomes of Foc1 and Foc4, respectively. It was calculated that approximately 83.78% and 79.90% of genes in Foc1 were transcribed at vegetative stage and 48 h post inoculation, respectively, which were comparable with 86.06% and 81.08% of that in Foc4 (Table 1). Among these expressed genes, 2,101were significantly up regulated and 4,153 were markedly down regulated in Foc1, while 2,410 and 5,642 were significantly up- regulated and down-regulated in Foc4, respectively (Figure 4). To confirm the accuracy of the RNA-seq result, 15 Foc genes were chosen randomly for real time quantitative PCR (qPCR). These genes were involved in signaling, biosynthesis, metabolism and pathogenesis, or were hypothetical proteins, and included up and down regulated genes as well as unaffected genes. The qPCR results are generally consistent with the variation in transcript levels determined by RNA-seq, suggesting the reliability of the RNA-seq data (Table S12 in File S1).

**Figure 5:**
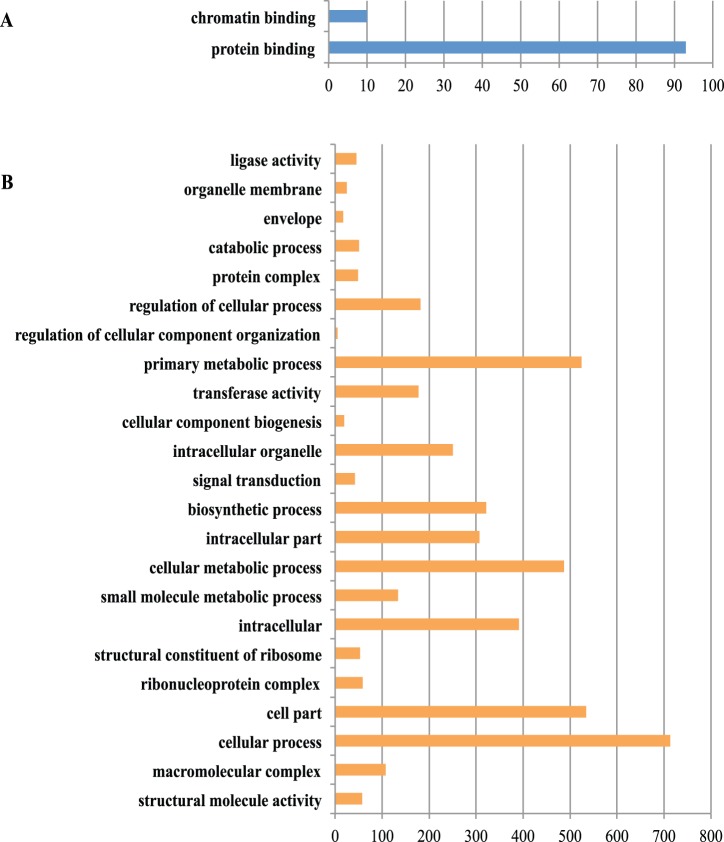
Gene Ontology (GO) functional annotation of differentially expressed genes (DEGs) in *Fusarium oxysporum* f. sp. *cubense* race 1 (Foc1). (**A**) Up-regulated genes in Foc1 could be only enriched into 2 groups. (**B**) Down-regulated genes in Foc1 could be enriched into three main GO categories and 23 groups on GO.level2 and GO.level3, 10 groups in biological process, 8 in cellular component, and 4 in molecular function. The X- axis represents the number of genes in a functional group.

**Figure 6:**
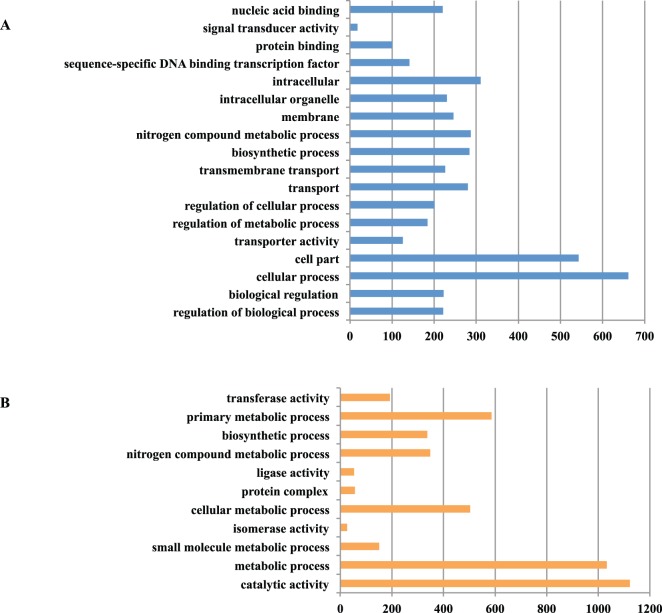
Gene Ontology (GO) functional annotation of differentially expressed genes (DEGs) in *Fusarium oxysporum* f. sp. *cubense* race 4 (Foc4). (**A**) Up-regulated genes in Foc4 could be enriched into three main GO categories and 18 groups on GO.level2 and GO.level3, 9 groups in biological process, 4 in cellular component, and 5 in molecular function. (**B**) Down-regulated genes in Foc1 could be enriched into 11 groups on GO.level2 and GO.level3, 6 groups in biological process, 1 in cellular component, and 4 in molecular function. The X- axis represents the number of genes in a functional group.

To provide a general view on the functions and processes that change in Foc1 and Foc4 at 48 h post inoculation to ‘Brazil’ banana, all the differentially expressed genes were classified into different functional categories. Up-regulated genes annotated in Gene Ontology (GO) for Foc1 could be only enriched into 2 functional groups “protein binding” and “chromatin binding”, while down-regulated genes in Foc1 were enriched into 23 groups on GO.level2 and GO.level3 (Figure 5, Table S13 in File S1). The most common categories for the down-regulated genes were “cellular process” and “cell part”, followed by “primary metabolic process”, “cellular metabolic process”, “intracellular” and “macromolecule metabolic process” (Table S13 in File S1). By contrast, up regulated genes annotated in GO for Foc4 could be enriched into 18 functional groups, including 9 in biological process, 4 in cellular component, 5 in molecular functions (Figure 6). Within biological process, “cellular process” (GO:0009987) with 661 genes, “nitrogen compound metabolic process” (GO:0006807) with 215 genes, “biosynthetic process” (GO:0009058) with 213 genes and “transport” with 210 genes were predominant. In the category of cellular component, the four groups were “membrane” (GO:0016020), “cell part” (GO:0044464), “integral to membrane” (GO:0016021) and “membrane part” (GO:0044425). Meanwhile, down-regulated genes annotated in GO were grouped into 11 groups on GO.level2 and GO.level3. The most frequently represented categories were “catalytic activity” and “metabolic process”, followed by “primary metabolic process”, “cellular metabolic process”, “nitrogen compound metabolic process” and “biosynthetic process” (Table S14 in File S1).

**Figure 7:**
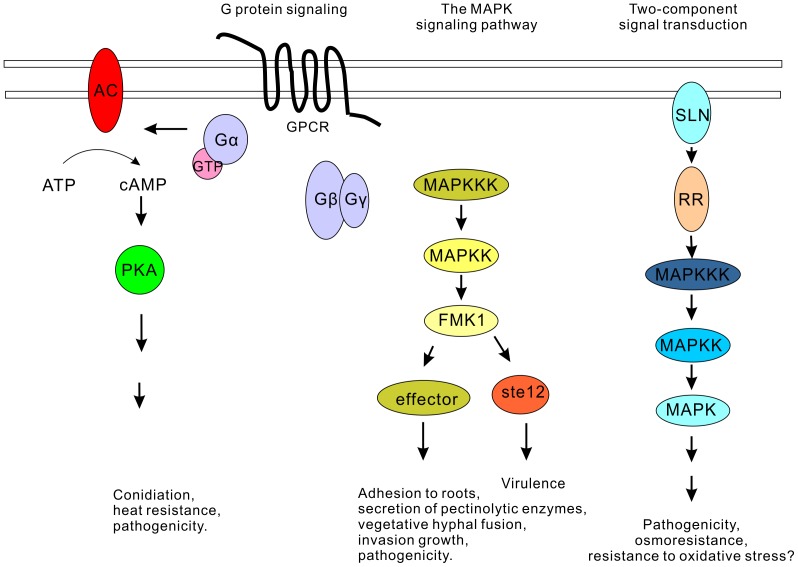
Schematic representation of signaling pathways activated in *F. oxysporum* f. sp. *cubense* race 4 isolate (Foc4) during infection of the banana variety ‘Brazil’. FGA1 (Gα) mediated G protein signaling, the FMK1-controlled mitogen-activated protein kinase signaling pathway and the pathogenicity associated two-component signal transduction system might be activated in Foc4. GPCR, G protein coupled receptor; Gα, G protein alpha subunit; Gβ & Gγ, G protein beta and gamma subunits; GTP, guanosine triphosphate; AC, adenylate cyclase; cAMP, cyclic adenosine monophosphate; ATP, adenosine triphosphate; PKA, protein kinase A; MAPKKK, mitogen-activated protein kinase kinase kinase; MAPKK, mitogen-activated protein kinase kinase; FMK1, mitogen-activated protein kinase; Ste12, transcription factor; SLN, histidine kinase; RR, response regulator. These pathways were characterized to associate with pathogenesis in fungal pathogens.

Upon infection *F. oxysporum* switches from a saprophytic to an infectious lifestyle, which probably includes the reprogramming of gene expression. Gene-expression data revealed a clear variation in the transcription levels of genes encoding putative GPCRs and G protein at 48 h following inoculation to the banana ‘Brazil’. In comparison with those at vegetative growth stage, transcript abundance of four putative GPCR genes (GPCR7, GPCR11, GPCR13 and GPCR20) and FGA1 (G protein alpha subunit) in Foc4 were significantly increased, while those of the counterparts in Foc1 were decreased or had no marked change (Table S15 in File S1). These imply that FGA1-mediated G protein signaling might be activated in Foc4 but not in Foc1 (Figure 7), and Foc1 and Foc4 might detect the signals from the host environment by different sensors. The similar result was reported in the entomopathogenic fungi *Metarhizium anisopliae* and *M. acridum,* which transcribed distinct GCPR genes on cuticles from locusts (the natural hosts) and cockroaches [127].

**Figure 8:**
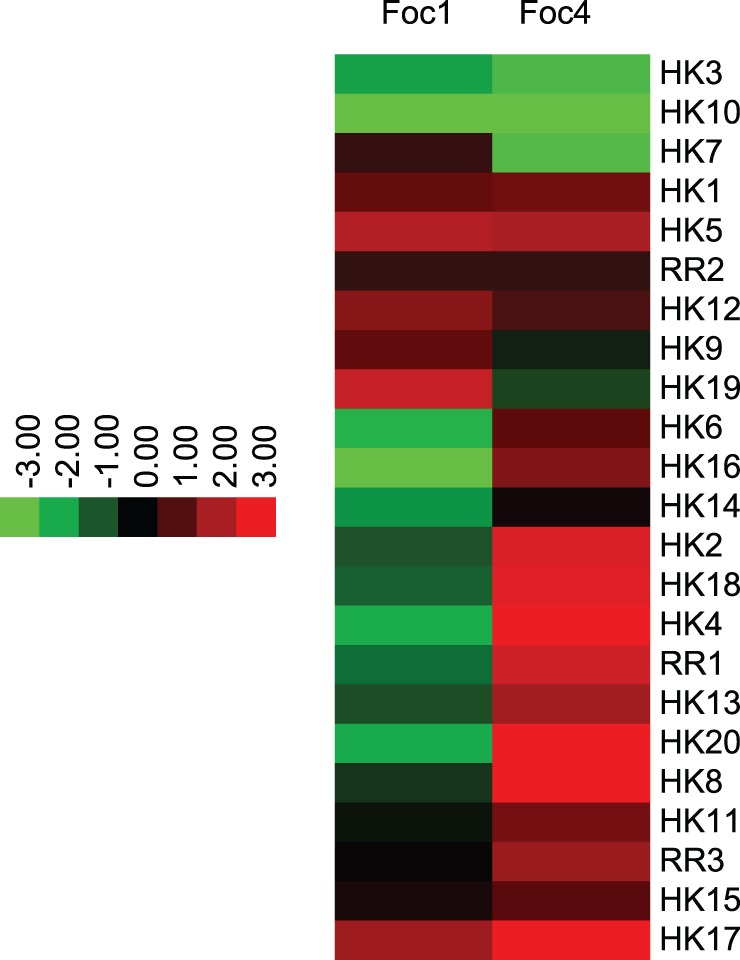
Differential gene expression profiling for the orthologous genes encoding histidine kinase from *Fusarium oxysporum* f. sp. *cubense* race 1 (Foc1) and race 4 (Foc4) infecting the banana ‘Brazil’. The Heat Map figures were generated using the log2 ratio of corresponding Foc1 (or Foc4) gene transcription data at 48 h post inoculation (48h_Reads Per Kilo bases per Million reads, 48h_RPKM) against Foc1 (or Foc4) 0h_RPKM data at vegetative growth stage, i.e. Log2 (48h-RPKM/0h_RPKM_). The figures show HK genes that are up regulated (red) and down regulated (green) relative to that at vegetative growth stage.

The two-component signal transduction systems seemed to play significant roles in recognition and adaption of the environmental change. RNA-seq data demonstrated that Foc1 and Foc4 could modulate the expression of different histidine kinase (HK) and response regulator (RR) genes (Figure 8). Relative to the vegetative growth stage, the more HKs were transcriptionally up regulated in Foc4 (9 HKs) than in Foc1 (4 HKs) at 48 h post inoculation to the banana ‘Brazil’, while less HK genes were down regulated in Foc4 (3 HKs) than in Foc1 (7 HKs) at 48 h post inoculation to the banana ‘Brazil’. Meanwhile, the expression of two RR genes was induced in Foc4, whereas none were affected in Foc1. These imply that more two-component signal transduction systems might be activated in Foc4 than in Foc1 during interaction between Foc4 and the banana host ‘Brazil’. Of particular note, the counterpart of the virulence-associated genes CaSLN [111] and MoSLN [106] in Foc4 was transcriptionally induced (Table S4 in File S1), but that in Foc1 was repressed, suggesting that the pathogenicity associated two-component signaling could be activated (Figure 7).

The protein kinases participate in a variety of cell processes and signal pathways including metabolism, cell signaling, protein regulation and other cellular pathways. We observed the variations in the expression of kinase genes between Foc1 and Foc4. Relative to the vegetative growth stage, Foc4 up-regulated more the protein kinase genes of AGC, CAMK and STE families than Foc1 during exposed to banana ‘Brazil’ (Figure S1).

Following the signal transduction, the distinct TFs would be activated. Inspecting the transcription of TF genes, we found that more TF genes of C2H2, MYB, bHLH, bZIP, Winged helix repressor DNA-binding and nucleic acid-binding (OB-fold) families were transcriptionally induced in Foc4 than in Foc1 at 48 hpi in comparison with the vegetative growth stage (Figure S2). The invasion growth pathway is controlled by the mitogen-activated protein kinase FMK1 and largely depends on the transcription factor Ste12 [11], which mediates distinct outputs downstream of the FMK1 cascade [126]. Although the expression of FMK1 was not induced in Foc1 and Foc4 at 48 h post inoculation as compared to that at vegetative growth stage, the transcripts level of *Ste12* was increased in Foc4 but not in Foc1, indicating the invasion growth pathway was activated in Foc4 but not in Foc1 at pre-infection stage (Figure 7).

RNA-seq data also revealed a clear variation in expression of carbohydrate-active enzyme coding genes including glycoside hydrolase (GH) family, polysaccharide lyases (PL), glycosyltransferases (GT) family, carbohydrate esterases (CE) family and carbohydrate-binding modules (CBM) family (Figure S3 & Figure S4). Compared to vegetative growth stage, the major of PL genes were induced after inoculated to banana ‘Brazil’ for 48 h (Figure S3). These imply that both Foc isolates could employ different carbohydrate-active enzymes to adapt the different nutrient conditions.

In Foc, the biosynthesis of secondary metabolites might be affected by the environmental change. 23 of backbone genes in Foc1 were transcribed at vegetative growth stage, while the number of expressed backbone genes reduced to 18 at 48 h post inoculation. Similarly, the number of expressed backbone genes in Foc4 decreased from 35 at vegetative growth stage to 29 at 48 h post inoculation. Moreover, relative to vegetative growth stage, 2 backbone genes were transcriptionally repressed while 6 were activated in Foc1 at 48 h post inoculation. In contrast, 10 backbone genes were induced and 13 were suppressed in Foc4. These imply that the different nutrient conditions have impact on the biosynthesis of secondary metabolites in Foc (Table S6 in File S1). The similar results were reported in the rice pathogen *Fusarium fujikuroi* [84], in which the secondary metabolites biosynthesis was affected by nitrogen availability.

### Conclusions

In this study, we have revealed that two Foc isolates are closely related to the tomato vascular wilt pathogen Fol by the phylogenetic analysis. Also, we have identified clear distinctions in gene contents and transcriptional regulation between Foc1 and Foc4, which may lead to the latter having a wider biochemical repertoire available for infecting the banana ‘Brazil’. The Foc genomic sequences will accelerate our efforts towards discovering pathogenicity mechanisms in *F. oxysporum* f. sp. *cubense*. This will eventually lead to improvement of Fusarium wilt disease resistance in banana.

### Accession Numbers

The Whole-Genome Shotgun projects have been deposited at DDBJ/EMBL/GenBank under the accession number AMGP00000000 for Foc1 and AMGQ00000000 for Foc4, respectively. The version described in this paper is the first version, AMGP00000000 and AMGQ00000000.

All short-read data have been deposited into the Short Read Archive (http://www.ncbi.nlm.nih.gov/sra) under the accession number SRA058029 and SRA058030. Raw sequencing data of the transcriptome have been deposited in the Gene Expression Omnibus with the accession number GSE40581.

## Materials and Methods

### 1. Fungal Isolates

The isolates N2 (race 1) and B2 (race 4) of *F. oxysporum* f. sp. *cubense* were isolated from diseased rhizomes of the banana (*Musa* spp.) cultivars ‘Brazil’ (AAA group) and ‘Pisang Awak’ (ABB group), respectively, in Hainan of China. The isolates were routinely maintained on potato dextrose agar (PDA),and the conidia of both isolates in 20% glycerol solution were stored at −80°C until use.

### 2. Genome Sequencing

We employed a whole genome shotgun strategy and the next-generation sequencing technologies using Illumina GA Analyzer to sequence the genomes of Foc1 (isolate N2) and Foc4 (isolate B2). To decrease the risk of non-randomness, sequencing libraries were constructed with insert sizes of about 500 base pairs (bp), 2,000 bp, 5,000 bp and 10,000 bp for Foc4, and of about 500 bp and 10,000 bp for Foc1 (Table 1).

### 3. Gene Prediction

We predicted the protein coding genes in Foc1 and Foc4 using a combination of de novo-based and homology-based approaches, as well as transcript evidence. For *de novo* predictions, Augustus (Version 2.5.5) [128] trained using *F. graminearum* was employed to predict coding genes. For the homology-based prediction, the whole protein sequence of *F. oxysporum* f. sp *lycopersici* (Fol), *F. graminearum*, *F. verticillioides*, *Nectria haematococca* (its asexual name *F. solani*), *Magnaporthe grisea* were collected from the website of broad institute (http://www.broadinstitute.org) and mapped onto the Foc genomes using TblastN. Then, homologous genome sequences were aligned against the matching proteins using Genewise to define gene models of Foc1 and Foc4. In addition, RNA-seq data generated in this study were mapped to both Foc genomes using Tophat [129], and transcriptome-based gene structures were obtained by cufflinks (http://cufflinks.cbcb.umd.edu/). Finally, all gene evidences were combined together using GLEAN (http://sourceforge.net/projects/glean-gene/).

### 4. Gene Family Classification

A Treefam [130], [131] based gene family analysis was conducted to study the gene family evolution and estimate the divergent time of Foc with other sequenced fungus. Protein sequences of Foc1, Foc4, *F. oxysporum* f. sp *lycopersici*, *F. verticillioides*, *F. graminearum*, *Nectria haematococca*, *Magnaporthe grisea* were selected to involve in this analysis.

### 5. Prediction of Secondary Metabolites Biosynthetic Gene Clusters

We employed the web-based software SMURF [132] (www.jcvi.org/smurf/) to systematically predict clustered SMs genes based on their genomic context and domain content. The software firstly identify the backbone genes acting as catalysts in biosynthesis of SMs, including prenyltransferases (DMAT), nonribosomal peptide synthases (NRPSs), polyketide synthases (PKSs), hybrid NRPS-PKS enzymes (HYBRID), then other related genes responsible for the modification, transportation and transcriptional regulation of SMs which are often found in contiguous gene clusters.

### 6. Identification of Horizontal Gene Transfer (HGT) -derived Genes in Foc1 and Foc4

Horizontal gene transfer (HGT) -derived gene was identified by phylogenetic method. The sequences of all gain proteins were aligned to ‘nr’ database of NCBI by using BLASTp with E-value cut-off 1e-5. Initial filtering to BLAST result set coverage >60% to reduce the frequency of single-domain matches to multi-domain proteins. Then we calculated lineage probabilities index (LPI) that is key to the genome-wide identification of horizontally transferred candidates [133]. Organisms closely related to the query genome receive higher LPI scores than the distant ones, and groups of phylogenetically related organisms receive similar scores to each other, regardless of their abundance or scarcity in the reference database [133]. At last,according to the baseline phylogenetic tree of each genes,we considered the genes only existing in Foc1/Foc4 and having no hit in other Fusarium species as the candidate HGT genes of Foc1/Foc4.

### 7. RNA Isolation and cDNA Synthesis

Foc isolates N2 (Foc1) and B2 (Foc4) were each incubated in a 250 ml-flask with 150 ml of PDB (potato dextrose broth) on a rotary shaker at 28°C at 180 rpm for 7 d, and then the mycelia and spores were harvested. For inoculation treatment, the cultures of Foc1 and Foc4 were suspended in sterile water and then each inoculated to the roots of ‘Brazil’ banana plantlets for 48 h in the hydroponics system. The cultures without inoculation and that inoculated to ‘Brazil’ for 48 h were used to isolate total RNA. RNA isolation and reverse transcription for cDNA synthesis were carried out using Trizol reagent (Invitrogen, USA) and the PrimeScript RT reagent Kit with gDNA Eraser (Takara, Japan) according the manufactory’s direction.

### 8. Transcriptome Analysis

Transcriptome sequences are obtained from Illumina HiSeq 2000 sequencers. All the high quality sequences were aligned against the reference genome with the spliced aligner TopHat [129]. Reads mapped to the genome were used to calculate every gene’s expression in each sample. We used RPKM (Reads Per Kb per Million reads) to estimate expressions of genes so as to normalize the effects of different gene lengths and different total mapped reads among samples [134]. Differentially expressed genes (DEGs) were detected using the method described by Chen *et al*. [135]. This method is based on the Poisson distribution and normalization for differences in RNA output sizes and sequencing depths between samples. P-value was used to test the statistical significance and FDR (false discovery rate) to determine the threshold of P-value in multiple tests. The threshold with “FDR< = 0.05″ and “absolute value of log2Ratio> = 1″ was set to judge the significance of differences. We firstly mapped all DEGs to GO terms in the database, calculating gene numbers for every term, then used hypergeometric test to find significantly enriched GO terms in DEGs comparing to the genome background.

### 9. Quantitative Real Time PCR Verification

To verify the transcriptome data, real time quantitative PCR assays were performed to analyze the relative expression levels of the several genes (Table S12 in File S1) in the Foc cultures without inoculation and the Foc cultures inoculating to the banana ‘Brazil’ for 48 h. *Actin* gene was used to normalize the gene expression. The primers listed in Table S12 in File S1 were used to amplification of these genes. Every sample was run twice with three replicates, and results were calculated according the delta-delta-Ct [136].

## Supporting Information

Figure S1 **Expression profiling of the orthologous genes encoding kinase in** ***Fusarium oxysporum*** **f. sp.** ***cubense*** **race 1 (Foc1) and race 4 (Foc4) infecting the banana ‘Brazil’.** Expression of genes encoding kinases of AGC, STE, CAMK and CMGC families was included. The Heat Map figures were generated using the log2 ratio of corresponding Foc1 (or Foc4) gene expression data at 48 h post inoculation (48h_Reads Per Kilo bases per Million reads, 48h_RPKM) against Foc1 (or Foc4) 0h_RPKM data at vegetative growth stage, i.e. Log2 (48h-RPKM/0h_RPKM). The figure shows kinase genes that are up regulated (red) and down regulated (green) relative to that at vegetative growth stage.
(EPS)

Figure S2 **Expression profiling of transcription factor genes in** ***Fusarium oxysporum*** **f. sp.** ***cubense*** **race 1 (Foc1) and race 4 (Foc4) attacking the banana ‘Brazil’.** Transcription factors of C2H2, MYB, bHLH, bZIP, WING, OB fold protein families were included. The Heat Map figures were generated as described in Figure S1. The figure shows kinase genes that are up regulated (red) and down regulated (green) relative to that at vegetative growth stage.
(EPS)

Figure S3 **Expression profiling of genes encoding glycoside hydrolases (GH) and polysaccharide lyases (PL) in** ***Fusarium oxysporum*** **f. sp.** ***cubense*** **attacking the banana ‘Brazil’.** The Heat Map figures were generated by the method described in Figure S1. The figures show kinase genes that are up regulated (red) and down regulated (green) relative to that at vegetative growth stage.
(EPS)

Figure S4 **Expression profiling of genes encoding carbohydrate esterases (CE), glycosyltransferases (GT) and carbohydrate-binding modules (CBM) in** ***Fusarium oxysporum*** **f. sp.** ***cubense*** **attacking the banana ‘Brazil’.** The Heat Map figures were generated by the method described in Figure S1. The figures show kinase genes that are up regulated (red) and down regulated (green) relative to that at vegetative growth stage.
(EPS)

File S1 **Tables S1–S15.** Table S1 in File S1. Statistics of sequencing data. Table S2 in File S1. Statistics of the assembled sequence length. Table S3 in File S1. Fast evolution genes in the banana fungal pathogens Foc1 and Foc4. Table S4 in File S1. The putative virulence associated genes in the banana fungal pathogens Foc1 and Foc4. Table S5 in File S1. The number of putative virulence associated genes in the sequenced fungi. Table S6 in File S1. The predict backbone genes involved in the biosynthesis of secondary metabolites in the banana fungal pathogens Foc1 and Foc4. Table S7 in File S1. The 26 predicted secondary metabolite synthesis gene clusters in Foc1. Table S8 in File S1. The 30 predicted secondary metabolite synthesis gene clusters in Foc4. Table S9 in File S1. The number of genes encoding putative G-protein coupled receptors encoded in *F. oxysporum* f. sp. *cubense*. Table S10 in File S1. Families of protein kinases in Foc1 and Foc4. Table S11 in File S1. The number of transcription factors in Foc1 and Foc4. Table S12 in File S1. Comparison of expression patterns between RNA-seq expression and quantitative real time PCR. Table S13 in File S1. Molecular functional classification of the differentially expressed genes in Foc1 based on GO.level2 and GO.level3. Table S14 in File S1. Molecular functional classification of the differentially expressed genes in Foc4 based on GO.level2 and GO.level3. Table S15 in File S1. The transcript levels of putative G protein coupled receptor and G protein genes.
(XLSX)
