# Correction: Genome and Transcriptome Analysis of the Fungal Pathogen *Fusarium oxysporum* f. sp. *cubense* Causing Banana Vascular Wilt Disease

**DOI:** 10.1371/journal.pone.0117621

**Published:** 2015-01-28

**Authors:** The PLOS ONE Staff

There is an error in the Materials and Methods section of this paper. Section 6, “Identification of Horizontal Gene Transfer (HGT) -derived Genes in Foc1 and Foc4,” should not appear.

In addition, Lijia Guo is incorrectly designated as the corresponding author. The correct corresponding authors are: Junsheng Huang (H888111@126.com), Ming Peng (mmpeng_2000@yahoo.com), and Xiaodong Fang (fangxd@genomics.org.cn).
